# Differences Between Tibial or Malleolar Fracture Types and Union or Nonunion in Spatiotemporal and Kinematic Gait Parameters Throughout Healing: An Observational Study

**DOI:** 10.1007/s10439-025-03937-2

**Published:** 2025-12-21

**Authors:** Elke Warmerdam, Jan Laqua, Jan Kattanek, Bergita Ganse

**Affiliations:** Werner Siemens-Endowed Chair for Innovative Implant Development (Fracture Healing), Departments and Institutes of Surgery, Saarland University, Kirrberger Straße 100, 66421 Homburg, Germany

**Keywords:** Gait analysis, Motion capture, Orthopaedic surgery, Rehabilitation, Injury, Traumatology

## Abstract

**Purpose:**

Gait analyses are becoming increasingly relevant in digital medicine. For implementation in clinical practice, knowledge on differences between gait patterns of separate bone fracture types is required. The aim of this study was to compare longitudinal changes in gait of patients with proximal tibial, tibial shaft, and malleolar fractures, as well as nonunion.

**Methods:**

Patients with a proximal tibial, tibial shaft, or malleolar fracture requiring surgery were prospectively enrolled in this longitudinal observational study. A healthy control group received one measurement. Optical motion capture was used to obtain spatiotemporal gait parameters and kinematics at 6 weeks, 3 months, and 6 months after surgery.

**Results:**

In total, 73 patients (51.1 ± 16.9 years) and 43 controls (50.5 ± 17.7 years) were included. Only in malleolar fractures, all gait parameters had returned to normal after 6 months. Differences between fracture types at 6 weeks were found in step height (*P* = 0.01), knee range of motion (ROM, *P* < 0.001), and its asymmetry (*P* < 0.001). At 6 months, knee ROM was still lower in proximal tibial than tibial shaft and malleolar fractures (*P* = 0.04; 0.047). Tibial shaft fractures with and without nonunion differed in stance time (*P* = 0.007; 0.02) and its asymmetry (*P* = 0.007; 0.009) after 6 weeks and 6 months, but not at 3 months.

**Conclusions:**

When monitoring fracture healing with motion capture, differences between fracture types and their timely appearance should be considered.

**Trial Registration:**

The study was prospectively registered in the German Clinical Trials Register DRKS00025108.

**Supplementary Information:**

The online version contains supplementary material available at 10.1007/s10439-025-03937-2.

## Introduction

Gait analysis is a valuable tool to monitor recovery progress in patients after fractures of the lower limbs and ankles [1]. For the best possible interpretation of these data and to be able to predict healing problems and complications in the future, it is crucial to conduct clinical observational studies that identify the parameters most suitable for healing prediction in each patient collective and injury type.

Following a lower leg fracture, the gait pattern usually improves slowly [1]. These improvements occur mainly during the first 3 months after the injury, but the full return to normal gait patterns can last up to 1 year after injury [2–4]. Gait speed and asymmetry measures seem to be the most suited to monitor fracture healing [1]. When monitoring gait continuously with instrumented insoles during the daily life of patients following tibial fracture, the maximal force, the unloading slope of the ground reaction force curve and the number of steps per day showed significant improvements during the first and second 6-week periods [5]. Furthermore, the asymmetry of the maximal force decreased significantly during the first 6 weeks. The most important kinematic parameters to analyse appear to be the knee and ankle joint flexion angles [3]. In tibial fracture patients, knee kinematics in squatting and knee kinematics and kinetics during walking did not completely recover during the first 6 months [2].

Spatiotemporal gait parameters include the domains pace, rhythm, variability, and asymmetry [1]. Kinematic parameters are joint angles and the range of motion (ROM). These parameters are usually assessed either by 3D optical motion capture systems with or without markers attached to the patient [6, 7], by IMU-based motion capture systems [8], and more recently also by low-cost camera or smartphone-based motion capturing [9]. The gold standard as of today are still the marker-based motion capture systems that use several cameras and are installed in a motion laboratory [6].

Differences in the spatiotemporal and kinematic parameter trajectories during recovery between different fracture types have not yet been investigated and the most ideal parameters to monitor healing progress have not yet been identified. Therefore, the aim of this study was to assess and compare longitudinal changes in gait spatiotemporal and kinematic parameters of patients with (1) proximal tibial, (2) tibial shaft, and (3) malleolar fractures, as well as (4) patients developing tibial shaft fracture nonunion. It was hypothesized that the longitudinal trajectories of spatiotemporal and kinematic gait parameters take longer to recover for patients with proximal tibial fractures than those with tibial shaft or malleolar fractures, and that tibial union and nonunion can be distinguished early on. In addition, differences in the time to return to the values of healthy controls were hypothesized.

## Materials and Methods

Ethics approval was obtained from the Institutional Review Board of Saarland Medical Board (application number 30/21). Written informed consent was obtained before the start of the measurements. This study was performed according to the Declaration of Helsinki. The measurements were conducted at Saarland University Hospital between June 2022 and April 2024. This purely observational study did not intervene with the treatment. It was registered in the German Clinical Trials Register (DRKS-ID: DRKS00025108) on the 21st of April 2021.

### Participants

Patients with a recent tibial or malleolar fracture that required surgery were enrolled in this longitudinal study. The exclusion criteria were age younger than 18 years, inability to provide consent, pregnancy, other injuries or conditions that affect mobility. The patients were grouped according to their type of fracture based on the AO fracture classification into proximal tibial, tibial shaft, and malleolar fractures [10]. Those patients who developed nonunion of the fracture throughout the observation period were grouped in a separate nonunion group. Nonunion was defined as a lack of callus bridging on radiographs at 6 months after surgery as judged by an experienced orthopaedic surgeon. A healthy control group, for which the same exclusion criteria applied as for the patients, was included in this study.

### Measurements

**Fig. 1:**
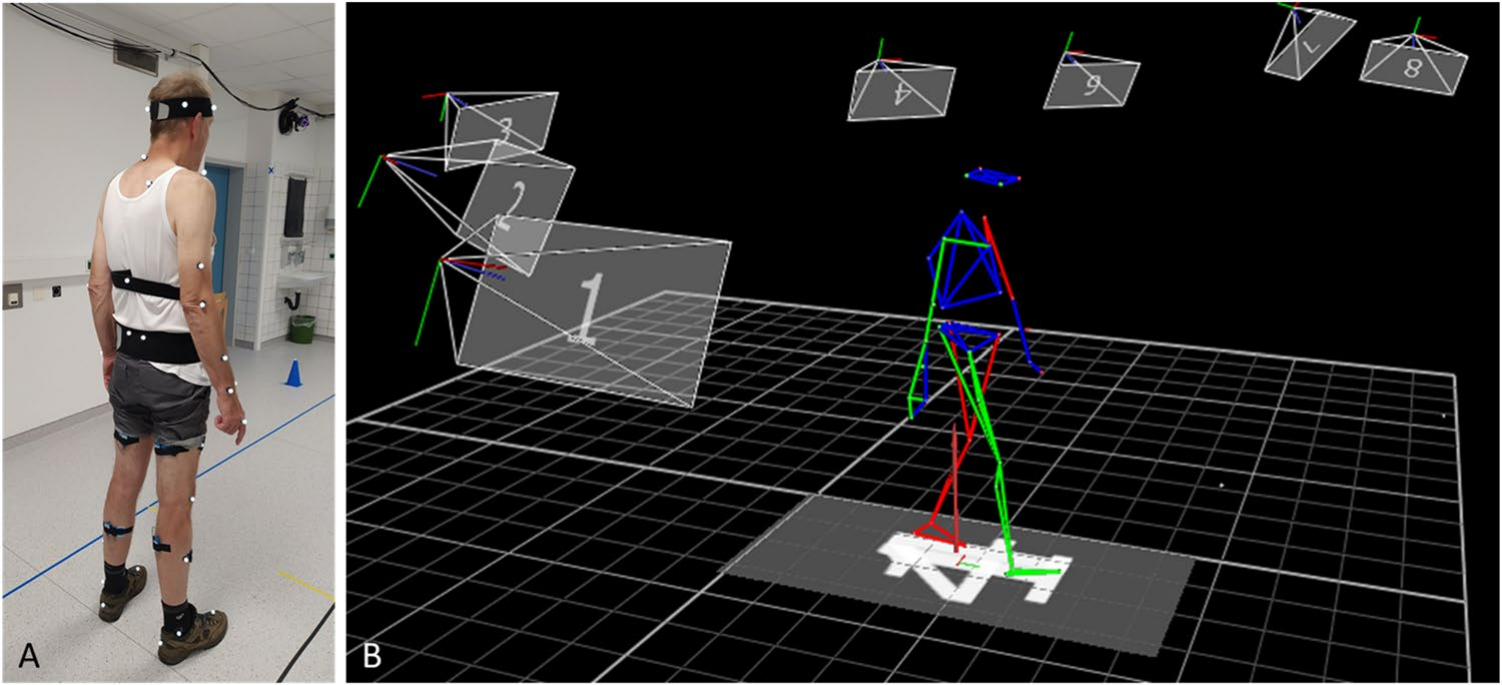
**A** Reflective markers on a healthy participant that are captured by the optical motion capture system. **B** A participant walking as captured by the software

Measurements took place at 6 weeks, 3 months and 6 months after surgery. The measurements were conducted in a motion laboratory next to the outpatient clinic where the patients had their clinical follow-up examinations. The motion lab is equipped with a 3D optical motion capture system with 13 cameras (Vero v2.2, Vicon, Oxford, United Kingdom). The reflective markers were placed according to the Vicon plug-in gait model, but only the lower body was taken into account for this study (Fig. 1). During a static calibration in neutral pose, additional markers were added on the medial side of the knee joint and on the malleolus medialis to improve the localisation of the knee and ankle joints. After the calibration measurement, participants were asked to walk in a straight line on the 10-m walkway with their own sport shoes on. Only during the 6 m in the middle of the walkway, data were captured, to make sure only steady state gait was recorded. This assessment was performed once during each clinical follow-up examination. The healthy participants received one measurement only. The position data of the markers were exported with the Vicon Nexus software (Nexus 2.12, Vicon, Oxford, United Kingdom).

### Data Analysis

Data analysis was performed with a custom-made MATLAB script (MATLAB R2021b, MathWorks, Natick MA, United States). Any gaps in the optical data were filled with an algorithm that is based on intercorrelations between markers [11]. This algorithm outperforms conventional gap-fill algorithms, especially in case of longer gaps and multiple missing markers [11]. Data were filtered with a bidirectional 4th order Butterworth filter with a cut-off frequency of 7 Hz. Gait speed was calculated by dividing the 6 m by the time it took to cover these 6 m, the middle of the pelvis (average of the four pelvic markers) was taken to determine the time as this position is an approximation of the centre of mass. The initial and final foot contacts during walking were extracted with a validated algorithm [12]. The timings of the initial and final contacts in combination with the position of the toe and heel markers were used to obtain the step length, step width, step height (of the middle of the foot), percentage stance time, stride time and step frequency. For the calculation of the joint angles, the segment orientation was obtained according to the International Society of Biomechanics recommendations [13]. The orientation of one segment related to the adjoint segment was used to calculate the joint angle. The maximum change in joint angle during a stride was defined as the range of motion (ROM) of that joint. The ROM of the ankle, knee and hip joint were calculated.

The asymmetry of several gait parameters was likewise calculated using Eq. 1. For the healthy controls, the left side was selected as the injured side in the equation.

1 $$\mathrm{Asymmetry}= \frac{(\mathrm{uninjured} \mathrm{side}-\mathrm{injured} \mathrm{side})}{(0.5*(\mathrm{uninjured} \mathrm{side}+\mathrm{injured} \mathrm{side}))}*100.$$

### Statistical Analysis

Statistical testing was performed with JASP (version 0.17.3, Amsterdam, The Netherlands). Differences in demographics between the groups was tested with a one-way ANOVA after testing for normal distribution using the Shapiro–Wilk test, if data were not normally distributed a Kruskal–Wallis test was used instead. The three fracture groups with union were compared with each other at the three different timepoints using one-way ANOVAs. Post hoc testing for the ANOVAs was done with Tukey corrections. The comparison of each fracture type with the healthy controls at each timepoint was performed with Mann–Whitney *U* tests. A nonparametric test was chosen because of the small group sizes. Likewise, the differences between the tibial shaft fractures with union and without union were analysed with a Mann–Whitney *U* test because of the small sample size at each timepoint. Significance was assumed at *P* < 0.05 for all tests.

## Results

A total of 73 patients with tibial or malleolar fractures were included in this study and divided in three groups based on the type of fracture. An additional group was formed by the patients who developed nonunion (Table 1). Forty-three healthy participants were included as the control group. Demographical values did not differ between the groups, except for weight which was higher in the patients with malleolar fractures compared to healthy controls (Tables 1, 2).

**Table 1:** Patient characteristics per fracture group with union.

	Proximal tibial fracture	Tibial shaft fracture (union)	Malleolar fracture	Healthy controls	*P* value proximal vs. tibial shaft vs. malleolar vs. controls (effect size)
*N* (% male)	18 (44)	12 (67)	36 (47)	43 (51)	
Age (years)	49.1 ± 15.5	46.8 ± 17.6	54.4 ± 16.3	50.5 ± 17.7	0.51 (0.02)
Height (m)	1.73 ± 0.11	1.78 ± 0.08	1.74 ± 0.09	1.74 ± 0.09	0.40 (0.03)
Weight (kg)	76.3 ± 14.7	83.8 ± 14.8	86.0 ± 17.9	75.8 ± 12.8	**0.02**^**a**^ (0.09)

^a^Significant difference between the weight of the malleolar fracture group and the healthy controls (*P* = 0.019)

**Table 2:** Patient characteristics of the tibial shaft fractures with and without union.

	Tibial shaft fracture (union)	Nonunion of tibial shaft fracture	*P* value tibial shaft union vs. nonunion (effect size)
*N* (% male)	12 (67)	7 (86)	
Age (years)	46.8 ± 17.6	50.9 ± 21.0	0.55 (0.18)
Height (m)	1.78 ± 0.08	1.83 ± 0.08	0.44 (0.23)
Weight (kg)	83.8 ± 14.8	87.4 ± 9.3	0.40 (0.25)

### Comparisons Between the Fracture Groups

**Fig. 2:**
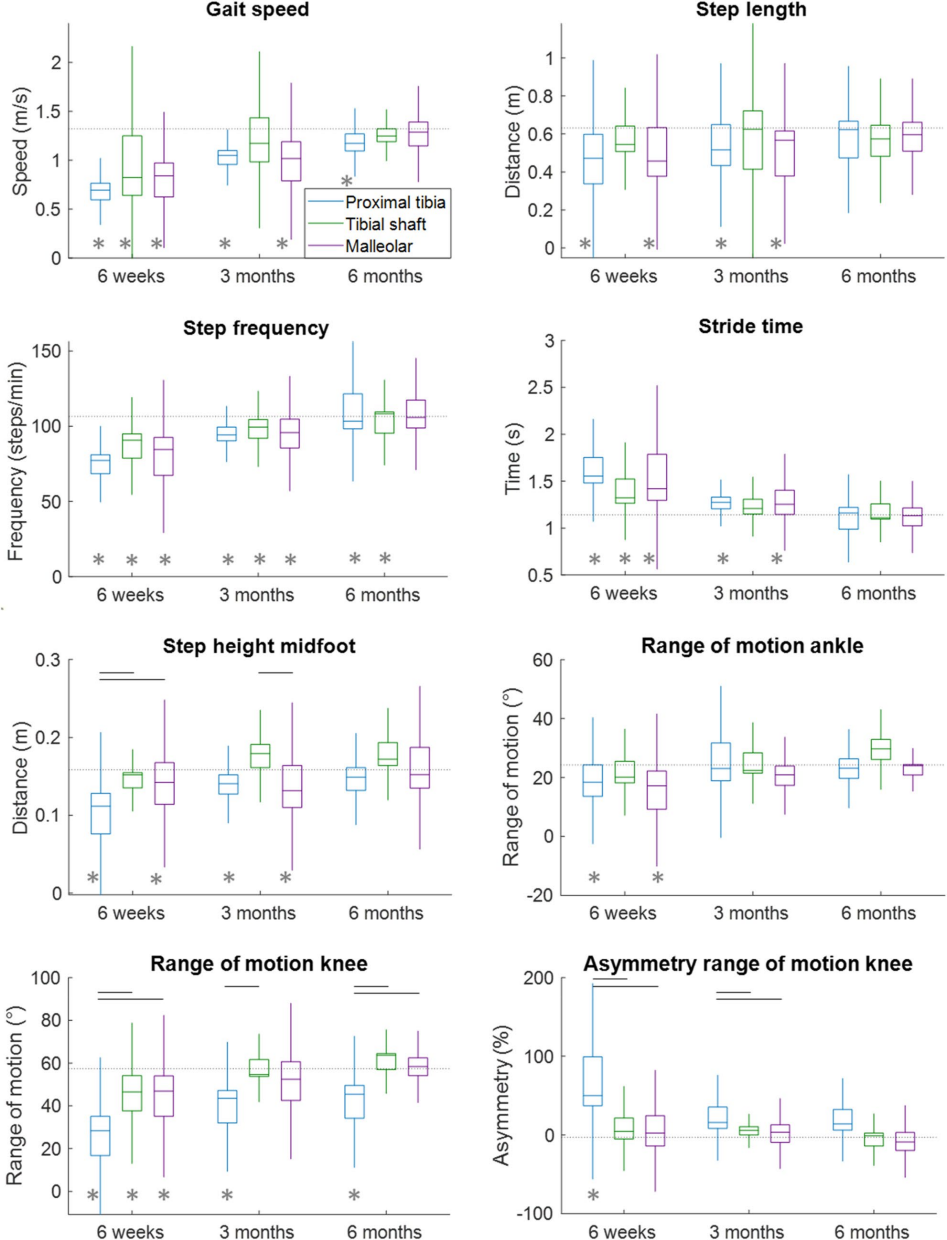
Boxplots of a subset of the gait parameters of the three fracture types with union at 6 weeks, 3 months and 6 months after surgery. Black horizontal lines at the top of the graph present significant differences between the fracture groups at that timepoint. The horizontal dotted line presents the average values of the healthy controls. The grey asterisks indicate significant differences between the fracture group and the healthy controls at the indicated timepoint. Boxplots of the other gait parameters can be found in Supplementary Fig. 1 and the average and SD values including the sample size can be found in Supplementary Tables 1–3.

**Fig. 3:**
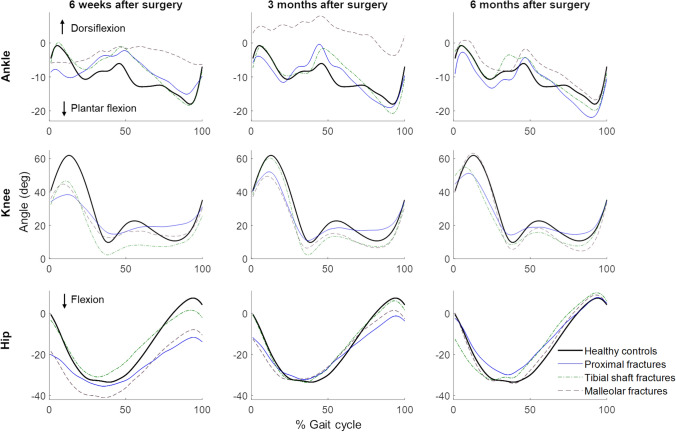
Joint angles during an average gait cycle per fracture group and healthy controls.

Three gait parameters differed significantly between the fracture types at 6 weeks and 3 months after surgery; the step height (*P* = 0.01), ROM of the knee (*P* < 0.001) and its asymmetry (*P* < 0.001, Table 3; Figs. 2, 3). At 6 months, only the ROM of the knee still differed significantly between the fracture types (*P* = 0.02). At 6 months, knee ROM was still lower in proximal tibial than tibial shaft fractures (*P* = 0.04), and between proximal tibial and malleolar fractures (*P* = 0.047). In most of the cases, the proximal tibial fracture group performed worse than the other groups.

**Table 3:** Results of the analyses regarding the differences between fracture types at 6 weeks, 3 months and 6 months after surgery.

	Six weeks *P* values (effect size)	Three months *P* values (effect size)	Six months *P* values (effect size)
Gait speed (m/s)	0.11 (0.08)	0.12 (0.09)	0.36 (0.08)
Step length (m)	0.34 (0.05)	0.58 (0.03)	0.76 (0.02)
Step width (m)	0.63 (0.02)	0.56 (0.03)	0.94 (0.01)
Step height (m)	**0.01 (0.17)**^**a (p=0.02), b(p=0.03)**^	**0.005 (0.22)**^**c (0.003)**^	0.19 (0.12)
Stance time (%)	0.51 (0.03)	0.46 (0.04)	0.62 (0.04)
Stride time (s)	0.16 (0.07)	0.40 (0.04)	0.88 (0.10)
Step frequency (steps/min)	0.08 (0.10)	0.41 (0.04)	0.80 (0.02)
ROM ankle (°)	0.56 (0.02)	0.19 (0.09)	0.10 (0.20)
ROM knee (°)	** < 0.001 (0.27)**^**a (p=0.005), b(p=0.001)**^	**0.02 (0.20)**^**a (p=0.018)**^	**0.02 (0.33)**^**a (p=0.043), b (p=0.047)**^
ROM hip (°)	0.28 (0.05)	0.53 (0.04)	0.08 (0.23)
Asymmetry step length (%)	0.92 (0.00)	0.93 (0.00)	0.96 (0.00)
Asymmetry step height (%)	0.37 (0.04)	0.33 (0.05)	0.19 (0.12)
Asymmetry percentage stance time (%)	0.11 (0.09)	0.40 (0.04)	0.15 (0.14)
Asymmetry ROM ankle (%)	0.23 (0.06)	0.29 (0.07)	0.62 (0.05)
Asymmetry ROM knee (%)	** < 0.001 (0.32)**^**a (p=0.004), b (p<0.001)**^	**0.010 (0.24)**^**a (p=0.43), b (p=0.010)**^	0.07 (0.26)
Asymmetry ROM hip (%)	0.53 (0.03)	0.65 (0.03)	0.28 (0.12)

*P* values and effect sizes are presented; significant values are shown in bold

*ROM* range of motion

^a^Significant difference between proximal tibial and tibial shaft fractures

^b^Significant difference between proximal tibial and malleolar fractures

^c^Significant difference between tibial shaft and malleolar fractures

### Comparisons with Healthy Controls

Six weeks after the fracture, the majority of the gait parameters showed significantly lower or longer (temporal parameters) values compared to those of the healthy control group (Figs. 2, 3; Supplementary Tables 4–6). From the 16 parameters, 13 were worse for the proximal tibial group, seven for the tibial shaft group and 11 for the malleolar group. Three months after the fracture, the number of parameters that significantly differed between the fracture groups and the healthy controls decreased further: 11, 3 and 8 parameters from, respectively, proximal tibia, tibial shaft and malleolar fractures were worse than those of healthy controls. This number decreased further to five, two and zero significantly different gait parameters at 6 months after surgery for, respectively, proximal tibia, tibial shaft and malleolar fractures. Thus, in malleolar fractures, all gait parameters returned to normal after 6 months. In the tibial shaft fracture patients, only the step frequency and the asymmetry of the step length remained significantly lower and greater, respectively. The same parameters also still differed in the proximal tibial fracture group, and in addition to these, knee ROM and its asymmetry, and the asymmetry of the stance time were worse compared to the control group. See the Supplementary Tables 4–6 for the *P* values.

### Comparisons Union vs. Nonunion

**Fig. 4:**
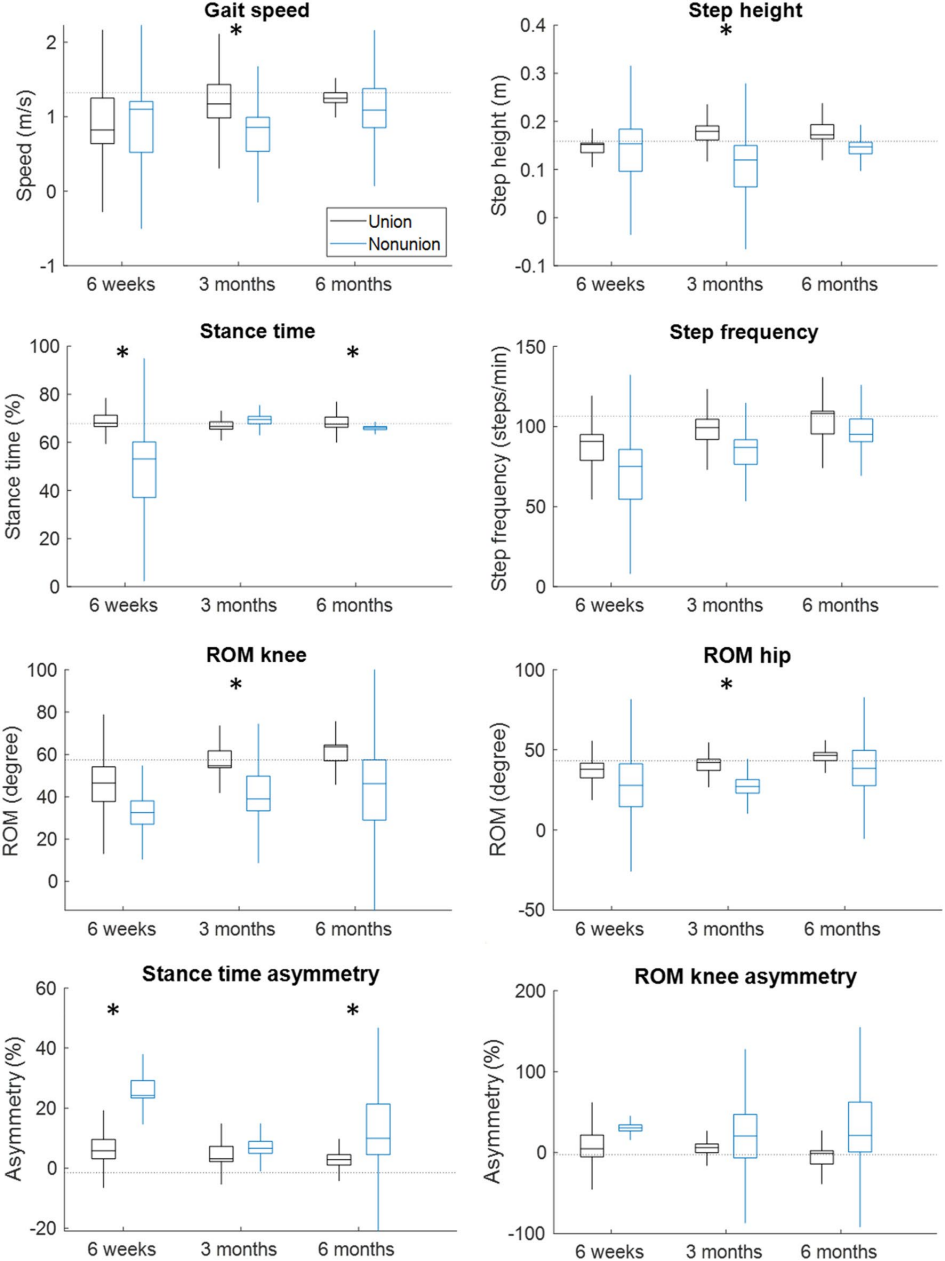
Boxplots of a subset of the gait parameters of the tibial shaft fracture union (black) and nonunion (blue) group at 6 weeks, 3 months and 6 months. Significant differences are presented with the asterisks at the top of the graph. The horizontal dotted line indicates the average results of the healthy control group. Boxplots of the other gait parameters can be found in the Supplementary Fig. 2. Average and SD values can be found in Supplementary Table 7.

The percentage stance time (*P* = 0.007) and its asymmetry (*P* = 0.007) were significantly shorter and greater, respectively, in the nonunion group compared to the union group 6 weeks after surgery. Similar results of the same parameters were observed at 6 months after surgery (*P* = 0.02 and 0.009). Paradoxically, at 3 months after surgery, different parameters showed significantly worse values for the nonunion group, namely gait speed (*P* = 0.02), step height (*P* = 0.02), ROM of the knee (*P* = 0.004) and ROM of the hip (*P* = 0.03) (Table 4; Fig. 4).

**Table 4:** Comparison between the tibial fracture union and nonunion group.

	Six weeks *P* values (effect size)	Three months *P* values (effect size)	Six months *P* values (effect size)
Gait speed (m/s)	0.89 (−0.09)	**0.02 (**−**0.74)**	0.54 (−0.27)
Step length (m)	0.81 (−0.13)	0.07 (−0.59)	0.43 (−0.33)
Step width (m)	0.22 (−0.53)	0.61 (0.19)	0.54 (0.27)
Step height (m)	0.57 (−0.27)	**0.02 (**−**0.74)**	0.05 (−0.73)
Stance time (%)	**0.007 (**−**1.00)**	0.33 (−0.33)	**0.02 (**−**0.87)**
Stride time (s)	0.81 (−0.13)	1.00 (0.00)	0.18 (0.53)
Step frequency (steps/min)	0.81 (0.13)	1.00 (0.00)	0.18 (0.53)
ROM ankle (°)	0.15 (−0.73)	0.15 (−0.48)	0.11 (−0.75)
ROM knee (°)	0.31 (−0.55)	**0.004 (**−**0.91)**	0.11 (−0.75)
ROM hip (°)	0.64 (−0.27)	**0.03 (**−**0.70)**	0.73 (−0.20)
Asymmetry step length	0.57 (0.27)	0.05 (0.63)	1.00 (0.00)
Asymmetry step height	0.29 (0.47)	0.07 (0.59)	0.33 (−0.40)
Asymmetry percentage stance time	**0.007 (1.00)**	0.09 (0.56)	**0.009 (0.93)**
Asymmetry ROM ankle	0.77 (0.18)	0.61 (0.19)	0.34 (0.50)
Asymmetry ROM knee	0.23 (0.64)	0.44 (0.29)	0.20 (0.63)
Asymmetry ROM hip	0.23 (0.64)	0.39 (0.30)	0.73 (0.20)

*P* values and effect sizes are presented; significant differences are shown in bold. Mean values and group size can be found in Supplementary Material

*ROM* range of motion

## Discussion

This study showed that the three gait-related parameters step height, knee ROM and its asymmetry differed between the analysed fracture types throughout healing. This finding was mainly caused by a slower recovery of the proximal tibial fractures as they performed worse than the other fracture types. In malleolar, but not in tibial fractures, all gait parameters had returned to normal after 6 months. The comparison of the patient groups with the healthy controls showed reductions in the differences over time. At 6 months after fracture, mainly the proximal tibial fractures still showed worse gait performance compared to the controls. Patients developing nonunion of a tibial shaft fracture showed a worse gait pattern compared to patients with union in several parameters already from 6 weeks on. Tibial shaft fractures with union improved the fastest up to 3 months after surgery. This observation is likely influenced by the implant type used, with patients receiving a nail were allowed immediate weight bearing.

The step height, knee joint ROM and its asymmetry showed differences between the fracture types analysed in this study. In most cases, it was the proximal tibial shaft group that performed worse than the other groups. The knee joint surface was injured in all patients with a proximal tibial fracture. Associated with this, pain or swelling in the knee joint could be the reason for smaller movements in the knee joint resulting in lower step height and smaller ROM. This finding is in line with a study by Li et al. [14] who showed that patients with more complex fractures and those treated with open reduction and internal fixation (ORIF) were more likely to show a decreased knee ROM 1 year after surgery. In addition, tibial eminence involvement with tibial plateau fractures has been shown to predict slower recovery and worse postoperative knee ROM [15]. The ankle or knee ROM most often showed the largest asymmetry values. Thus, these values seem to be the most sensitive, and it would be beneficial if they could be determined in clinical practice. It appears that the ROM-asymmetry parameters are more sensitive than spatiotemporal asymmetry parameters to monitor progress. The remaining differences in knee ROM between the groups are likely clinically relevant, as the ROM of lower limb joints has a profound effect on the quality of life of fracture patients [16, 17]. Unfortunately, the ROM parameters are more difficult to measure outside of the lab. Most spatiotemporal parameters can be easily measured with a wearable sensor (e.g., inertial measurement unit, IMU) [8], but to measure ROM of a joint, at least two IMUs with regular calibration procedures are required [8, 18], making it less feasible to measure ROM during routine clinical care.

Patients who developed a nonunion of the fracture already performed worse in several gait parameters compared to patients with union from 6 weeks on. This indicates that gait analysis may help the clinician to predict nonunion with more certainty and to choose to intervene earlier. By detecting patients at risk of a nonunion early on, additional non-invasive therapy, such as low-intensity pulsed ultrasound [19, 20], extracorporeal shockwave therapy [21], and pulsed electromagnetic fields [22] to stimulate fracture healing can be started earlier to potentially prevent nonunion and to facilitate fracture healing [23].

Motion capture systems used to be expensive and time-consuming, as well as difficult to use for laypersons or in a clinical context. With the emergence of pose estimation [24] and thereby markerless systems [7, 25], and smartphone applications that can deliver at least some parameters [26, 27], totally new opportunities arise for clinical application. While the accuracy of the separate options may vary, the parameters determined in this study are useful independent of the technology and platform used to assess these data.

Smart implants to treat bone fractures that are equipped with sensors for continuous monitoring are currently being developed for clinical use [28]. Among the sensor types that could be embedded in a smart implant are IMUs and force sensors. Concerning the parameters analysed in this study, sensors in an implant would not allow for gait asymmetry analysis, as usually only one leg is fractured and would receive a smart implant. This means that implant-based movement analysis would need to rely on the data from one leg only. However, temporal gait parameters, such as stance time, swing time, stride time and stride frequency could be easily assessed by such sensors. Spatial parameters could also be estimated with IMU data, but these algorithms require some assumptions which could make the results slightly less accurate. Based on the findings of this study, to predict nonunion, stance time seems to be the most useful of these parameters.

While it is certainly not possible to continuously monitor all spatiotemporal and kinematic parameters during the daily life of patients, smart insoles equipped with pressure sensors, and IMUs are another option for patient monitoring [5]. They could be used in addition to an implant, or as a sole data source in conservative treatment. When analysing the pressure sensor data, it needs to be considered that the trajectory of the force curve during the stance phase depends on factors such as the surface type [29], slope [30], as well as anthropometric parameters [31]. Similar to IMU systems, also smart insoles could be employed to assess the stance time that appears to be the best parameter to predict nonunion based on the findings of the present study.

### Limitations

This study has several limitations. The sample size was relatively small and the group sizes differed between timepoints, because of early drop-outs or missed appointments. Besides that, there were significant differences in gait between fracture types, but it is also possible that within the individual fracture type groups, there are differences due to the variations in fracture severity [32]. Fracture severity was not taken into account in this study. The included patients were a rather heterogenic group; however, it also reflects the actual fracture population. Future studies could also analyse sex and age differences, however, in this study, the group size would have become too small to do this. The 10 m walkway of which only the middle 6 m-section was taken into account for analysis is rather short and not comparable to daily life walking.

## Conclusion

There were differences in the determined gait parameters between fracture types throughout fracture healing, but most gait parameters did not differ between the groups. However, the differences between the fracture types should be taken into account. The gait pattern of proximal tibial fractures required the most time to return to values of healthy control subjects and some gait parameters did not return to these levels within 6 months. Patients who developed a nonunion of the tibial shaft fracture already showed a worse gait performance from 6 weeks onward, which indicates that the use of gait analysis has potential to help the clinicians detect patients at risk of developing nonunion earlier on. The results should be interpreted with caution before they have been confirmed in larger studies.

## Supplementary Information

Below is the link to the electronic supplementary material.

Supplementary file1 (PDF 389 kb)

## Data Availability

The data set analysed during the present study is available from the corresponding author on reasonable request. Access may be granted based on a collaboration agreement. The requesting institution needs to fall within the eligibility criteria of German Data Protection Law. All authors agree to the publication of this manuscript.
