# Supplementary material for: Differences Between Tibial or Malleolar Fracture Types and Union or Nonunion in Spatiotemporal and Kinematic Gait Parameters Throughout Healing: An Observational Study
Source: Ann Biomed Eng. 2025 Dec 21;54(4):1164–74. doi: 10.1007/s10439-025-03937-2 (PMC13035564; doi:10.1007/s10439-025-03937-2)
Supplement: Supplementary file 1 — Supplementary file1 (PDF 389 kb) [file 10439_2025_3937_MOESM1_ESM.pdf]

Supplementary material

Title: Differences between tibial or malleolar fracture types and union or nonunion in spatiotemporal and kinematic gait parameters throughout healing: An observational study

Annals of Biomedical Engineering

Elke Warmerdam[1], Jan Laqua[1], Jan Kattanek[1], Bergita Ganse[1]

1 Werner Siemens-Endowed Chair for Innovative Implant Development (Fracture Healing), Departments and Institutes of Surgery, Saarland University, Kirrberger Straße 100, 66421 Homburg, Germany

Corresponding author:

Elke Warmerdam

Elke.warmerdam@uni-saarland.de

Supplementary table 1. Mean values, standard deviations and group size of the three fracture type groups with union at six weeks post-surgery.

Week 6	Mean (SD) of proximal tibial fractures	Mean (SD) of tibial shaft fractures	Mean (SD) of malleolar fractures
Gait speed (m/s)	0.69 (0.25), n=16	0.94 (0.38), n=11	0.79 (0.28), n=28
Step length (m)	0.47 (0.16), n=15	0.56 (0.14), n=10	0.49 (0.14), n=27
Step width (m)	0.12 (0.03), n=15	0.13 (0.04), n=10	0.12 (0.03), n=27
Step height of mid foot (m)	0.10 (0.03), n=15	0.15 (0.02), n=10	0.14 (0.04), n=27
Stance time (%)	65.5 (6.9), n=15	68.0 (5.8), n=10	64.2 (10.7), n=27
Stride time (s)	1.69 (0.39), n=15	1.41 (0.28), n=10	1.56 (0.36), n=27
Step frequency (steps/min)	73.5 (12.6), n=15	87.8 (15.7), n=10	80.2 (15.8), n=27
ROM ankle (°)	19.5 (8.1), n=16	21.0 (6.2), n=11	17.4 (11.4), n=23
ROM knee (°)	27.0 (13.3), n=16	44.7 (13.6), n=11	43.8 (13.6), n=23
ROM hip (°)	30.1 (9.2), n=16	36.7 (7.6), n=11	34.2 (13.1), n=24
Asymmetry step length	11.2 (53.9), n=15	3.9 (45.4), n=10	11.9 (55.3), n=26
Asymmetry step height	12.8 (41.9), n=15	6.4 (20.8), n=10	-3.0 (35.4), n=27
Asymmetry percentage stance time	16.5 (14.8), n=15	5.2 (7.9), n=10	15.0 (15.2), n=27
Asymmetry ROM ankle	37.6 (43.0), n=16	19.1 (36.2), n=11	63.2 (44.8), n=22
Asymmetry ROM knee	63.2 (44.8), n=16	15.0 (36.6), n=11	8.4 (30.2), n=23
Asymmetry ROM hip	24.6 (25.4), n=16	11.4 (19.5), n=11	20.0 (36.2), n=24

Supplementary table 2. Mean values, standard deviations and group size of the three fracture type groups with union at three months post-surgery.

Month 3	Mean (SD) of proximal tibial fractures	Mean (SD) of tibial shaft fractures	Mean (SD) of malleolar fractures
Gait speed (m/s)	1.02 (0.18), n=13	1.21 (0.31), n=9	1.01 (0.26), n=24
Step length (m)	0.52 (0.15), n=13	0.58 (0.17), n=9	0.52 (0.15), n=24
Step width (m)	0.12 (0.04), n=13	0.13 (0.03), n=9	0.12 (0.04), n=24
Step height of mid foot (m)	0.14 (0.02), n=13	0.18 (0.03), n=9	0.13 (0.04), n=24
Stance time (%)	67.2 (9.5), n=13	66.3 (4.8), n=9	69.3 (5.8), n=24
Stride time (s)	1.33 (0.20), n=13	1.23 (0.12), n=9	1.27 (0.18), n=24
Step frequency (steps/min)	91.8 (11.1), n=13	98.7 (9.4), n=9	96.3 (14.0), n=24
ROM ankle (°)	25.2 (9.5), n=12	25.1 (6.1), n=9	21.1 (4.3), n=18
ROM knee (°)	41.7 (11.1), n=11	57.0 (7.4), n=9	50.2 (13.7), n=18
ROM hip (°)	37.5 (7.6), n=11	41.2 (5.4), n=9	39.8 (8.3), n=18
Asymmetry step length	13.8 (27.3), n=13	7.2 (42.1), n=9	8.3 (51.0), n=24
Asymmetry step height	6.3 (13.4), n=13	-14.3 (31.6), n=9	1.6 (39.0), n=24
Asymmetry percentage stance time	8.8 (17.7), n=13	5.8 (7.0), n=9	3.4 (8.4), n=24
Asymmetry ROM ankle	3.7 (32.6), n=10	17.3 (27.3), n=9	21.4 (25.4), n=18
Asymmetry ROM knee	26.7 (30.9), n=10	3.0 (13.5), n=9	1.5 (15.9), n=18
Asymmetry ROM hip	8.1 (14.9), n=11	2.4 (11.7), n=9	9.0 (21.2), n=18

Supplementary table 3. Mean values, standard deviations and group size of the three fracture type groups with union at six months post-surgery.

Month 6	Mean (SD) of proximal tibial fractures	Mean (SD) of tibial shaft fractures	Mean (SD) of malleolar fractures
Gait speed (m/s)	1.16 (0.24), n=10	1.25 (0.17), n=6	1.31 (0.27), n=13
Step length (m)	0.57 (0.13), n=10	0.57 (0.09), n=6	0.60 (0.14), n=13
Step width (m)	0.12 (0.04), n=10	0.12 (0.05), n=6	0.11 (0.06), n=13
Step height of mid foot (m)	0.15 (0.03), n=10	0.17 (0.03), n=6	0.16 (0.03), n=13
Stance time (%)	66.1 (4.3), n=10	68.5 (3.5), n=6	66.6 (5.1), n=13
Stride time (s)	1.13 (0.23), n=10	1.15 (0.08), n=6	1.11 (0.13), n=13
Step frequency (steps/min)	110.3 (23.8), n=10	104.6 (7.3), n=6	109.6 (14.3), n=13
ROM ankle (°)	23.7 (7.8), n=8	29.5 (4.0), n=4	22.5 (3.2), n=11
ROM knee (°)	41.9 (14.2), n=7	60.7 (6.7), n=4	56.1 (10.7), n=11
ROM hip (°)	40.5 (6.2), n=7	46.2 (3.7), n=5	45.0 (3.6), n=11
Asymmetry step length	11.0 (35.5), n=10	11.9 (8.4), n=6	8.1 (28.3), n=13
Asymmetry step height	2.8 (10.3), n=10	-1.7 (9.3), n=6	-9.7 (26.3), n=13
Asymmetry percentage stance time	5.5 (6.6), n=10	-2.8 (2.1), n=6	4.6 (10.9), n=13
Asymmetry ROM ankle	15.8 (35.2), n=8	-6.8 (20.9), n=4	8.3 (42.4), n=11
Asymmetry ROM knee	21.0 (19.3), n=6	-5.9 (13.1), n=4	-5.7 (25.0), n=11
Asymmetry ROM hip	8.6 (11.9), n=7	-3.3 (12.2), n=5	1.0 (13.8), n=11

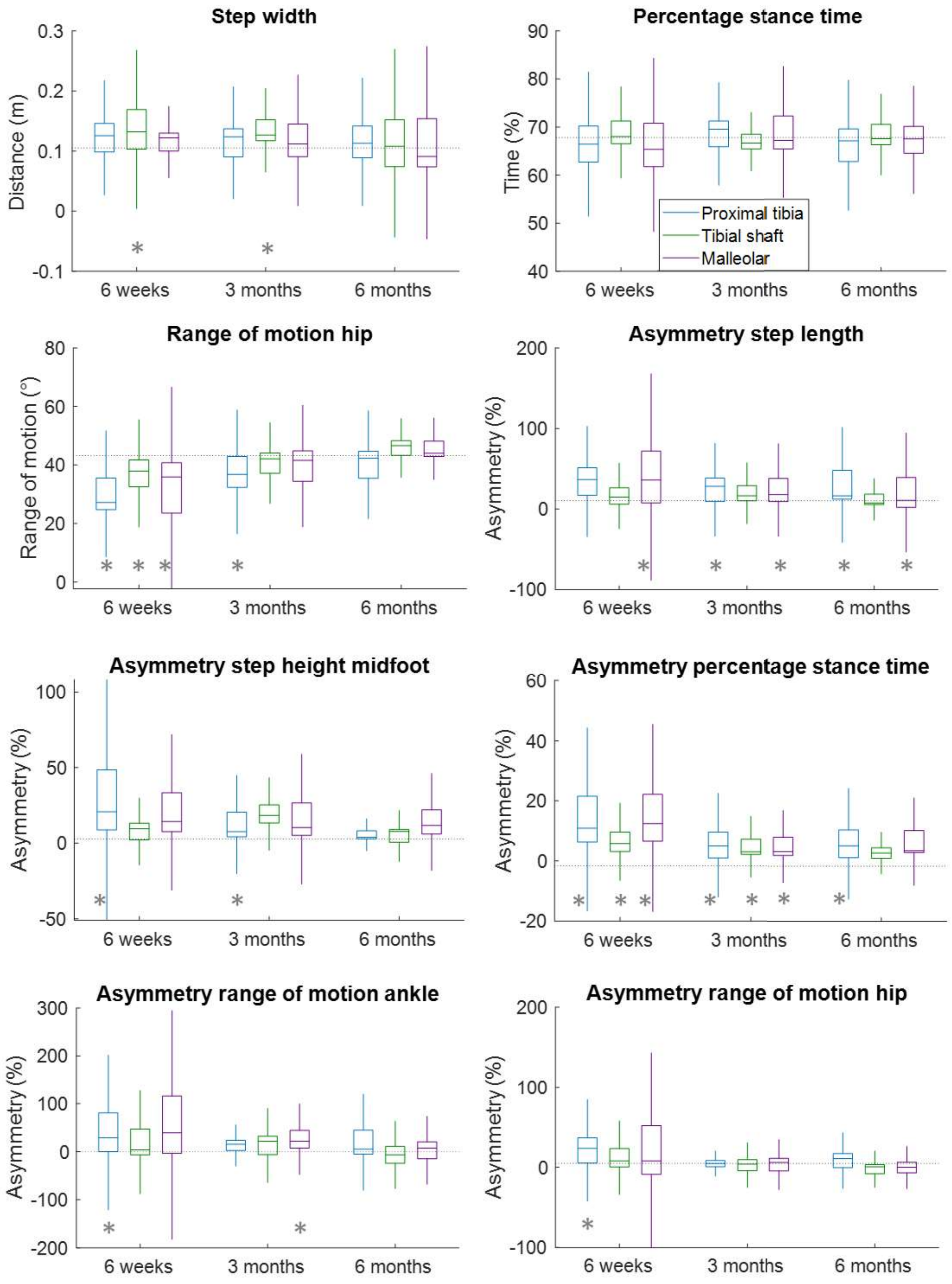

Supplementary figure 1. Boxplots of a subset of the gait parameters of the three fracture types at six weeks, three months and six months after surgery. The horizontal dotted line presents the values of the healthy controls. The grey asterisks indicate significant differences between the fracture group and the healthy controls at the indicated timepoint.

Supplementary table 4. P values and effect sizes of the comparison between fracture type and healthy controls at six weeks post-surgery.

Week 6	P values comparison proximal tibial fractures and controls (effect sizes)	P values comparison tibial shaft fractures and controls (effect sizes)	P values comparison malleolar fractures and controls (effect sizes)
Gait speed (m/s)	**< .001 (-0.91)**	**0.002 (-0.60)**	**< .001 (-0.86)**
Step length (m)	**< .001 (-0.59)**	0.118 (-0.32)	**< .001 (-0.55)**
Step width (m)	0.055 (0.34)	**0.028 (0.45)**	0.061 (0.27)
Step height of mid foot (m)	**< .001 (-0.82)**	0.063 (-0.38)	**0.008 (-0.38)**
Stance time (%)	0.536 (-0.11)	0.672 (0.09)	0.086 (-0.25)
Stride time (s)	**< .001 (0.98)**	**0.001 (0.66)**	**< .001 (0.85)**
Step frequency (steps/min)	**< .001 (0.78)**	**< .001 (0.99)**	**< .001 (0.86)**
ROM ankle (°)	**0.019 (-0.40)**	0.118 (-0.31)	**< .001 (-0.51)**
ROM knee (°)	**< .001 (-0.97)**	**< .001 (-0.63)**	**< .001 (-0.63)**
ROM hip (°)	**< .001 (-0.75)**	**0.007 (-0.51)**	**< .001 (-0.50)**
Asymmetry step length	0.512 (0.11)	0.864 (0.04)	**0.002 (0.45)**
Asymmetry step height	**< .001 (0.60)**	0.339 (0.20)	0.801 (0.04)
Asymmetry percentage stance time	**< .001 (0.92)**	**0.004 (0.57)**	**< .001 (0.76)**
Asymmetry ROM ankle	**0.003 (0.50)**	0.147 (0.29)	**0.002 (0.48)**
Asymmetry ROM knee	**< .001 (0.82)**	0.089 (0.33)	0.396 (0.13)
Asymmetry ROM hip	**0.001 (0.53)**	0.392 (0.17)	0.415 (0.12)

Supplementary table 5. P values and effect sizes of the comparison between fracture type and healthy controls at three months post-surgery.

Month 3	P values comparison proximal tibial fractures and controls (effect sizes)	P values comparison tibial shaft fractures and controls (effect sizes)	P values comparison malleolar fractures and controls (effect sizes)
Gait speed (m/s)	**< .001 (-0.67)**	0.276 (-0.23)	**< .001 (-0.60)**
Step length (m)	**0.013 (-0.45)**	0.706 (-0.08)	**0.007 (-0.39)**
Step width (m)	0.217 (0.23)	**0.049 (0.42)**	0.394 (0.13)
Step height of mid foot (m)	**0.006 (-0.50)**	0.123 (0.33)	**0.004 (-0.43)**
Stance time (%)	0.341 (0.18)	0.299 (-0.22)	0.879 (0.02)
Stride time (s)	**< .001 (0.66)**	0.061 (0.40)	**0.002 (0.46)**
Step frequency (steps/min)	**< .001 (0.99)**	**< .001 (1.00)**	**< .001 (1.00)**
ROM ankle (°)	0.975 (-0.01)	0.823 (0.05)	0.059 (-0.31)
ROM knee (°)	**< .001 (-0.81)**	0.691 (-0.09)	0.069 (-0.30)
ROM hip (°)	**0.013 (-0.49)**	0.299 (-0.22)	0.185 (-0.22)
Asymmetry step length	**0.008 (0.50)**	0.061 (-0.42)	**0.025 (0.33)**
Asymmetry step height	**0.006 (0.50)**	0.123 (-0.33)	0.093 (0.25)
Asymmetry percentage stance time	**0.002 (0.55)**	**0.001 (0.65)**	**0.002 (0.45)**
Asymmetry ROM ankle	0.405 (0.18)	0.138 (0.32)	**0.009 (0.43)**
Asymmetry ROM knee	**< .001 (0.70)**	0.152 (0.31)	0.409 (0.14)
Asymmetry ROM hip	0.905 (0.03)	0.725 (-0.08)	0.829 (0.04)

Supplementary table 6. P values and effect sizes of the comparison between fracture type and healthy controls at six months post-surgery.

Month 6	P values comparison proximal tibial fractures and controls (effect sizes)	P values comparison tibial shaft fractures and controls (effect sizes)	P values comparison malleolar fractures and controls (effect sizes)
Gait speed (m/s)	0.072 (-0.37)	0.376 (-0.22)	0.684 (-0.08)
Step length (m)	0.124 (-0.32)	0.180 (-0.34)	0.226 (-0.23)
Step width (m)	0.285 (0.22)	0.894 (0.03)	0.924 (0.02)
Step height of mid foot (m)	0.070 (-0.37)	0.394 (0.22)	0.744 (-0.06)
Stance time (%)	0.575 (-0.12)	0.828 (0.06)	0.930 (-0.02)
Stride time (s)	1.000 (-0.00)	0.784 (0.07)	0.808 (-0.05)
Step frequency (steps/min)	**< .001 (1.00)**	**< .001 (1.00)**	< .001 (1.00)
ROM ankle (°)	0.926 (-0.02)	0.069 (0.56)	0.611 (-0.10)
ROM knee (°)	**< .001 (-0.77)**	0.202 (0.40)	0.895 (0.03)
ROM hip (°)	0.304 (-0.25)	0.303 (0.30)	0.392 (0.17)
Asymmetry step length	**0.046 (0.41)**	**< .001 (0.93)**	0.018 (0.44)
Asymmetry step height	1.000 (0.00)	0.200 (-0.33)	0.217 (-0.23)
Asymmetry percentage stance time	**0.001 (0.63)**	0.645 (-0.12)	0.052 (0.36)
Asymmetry ROM ankle	0.317 (0.23)	0.551 (-0.19)	0.808 (0.05)
Asymmetry ROM knee	**0.001 (0.76)**	0.923 (-0.03)	0.297 (-0.21)
Asymmetry ROM hip	0.361 (0.22)	0.215 (-0.35)	0.292 (-0.21)

Supplementary table 7. Mean values, standard deviations and group size of the nonunion tibial shaft group at six weeks, three months and six months post-surgery.

	Mean (SD) of nonunion tibial fractures at six weeks	Mean (SD) of nonunion tibial fractures at three months	Mean (SD) of nonunion tibial fractures at six months
Gait speed (m/s)	0.89 (0.49), n=3	0.80 (0.28), n=6	1.09 (0.33), n=5
Step length (m)	0.51 (0.03), n=3	0.39 (0.11), n=6	0.46 (0.15), n=5
Step width (m)	0.12 (0.01), n=3	0.14 (0.02), n=6	0.13 (0.01), n=5
Step height of mid foot (m)	0.11 (0.04), n=3	0.09 (0.03), n=6	0.11 (0.02), n=5
Stance time (%)	61.5 (2.8), n=3	67.0 (1.8), n=6	66.2 (2.5), n=5
Stride time (s)	1.46 (0.08), n=3	1.50 (0.15), n=6	1.42 (0.08), n=5
Step frequency (steps/min)	82.2 (4.3), n=3	80.4 (7.5), n=6	84.7 (4.9), n=5
ROM ankle (°)	14.8 (2.0), n=2	19.9 (6.8), n=6	22.4 (4.1), n=4
ROM knee (°)	32.5 (7.8), n=2	40.3 (9.7), n=6	43.2 (19.3), n=4
ROM hip (°)	27.8 (19.0), n=2	29.8 (9.8), n=6	38.6 (13.2), n=4
Asymmetry step length	-3.0 (18.2), n=3	19.7 (18.7), n=6	8.4 (52.8), n=5
Asymmetry step height	17.4 (6.3), n=3	16.8 (11.7), n=6	-10.3 (24.6), n=5
Asymmetry percentage stance time	25.9 (4.0), n=3	15.6 (6.4), n=6	16.2 (6.8), n=5
Asymmetry ROM ankle	32.2 (52.3), n=2	20.0 (18.2), n=6	10.1 (20.7), n=4
Asymmetry ROM knee	30.3 (5.2), n=2	19.2 (29.4), n=6	31.4 (42.7), n=4
Asymmetry ROM hip	51.0 (52.0), n=2	15.7 (23.3), n=6	12.0 (27.6), n=4

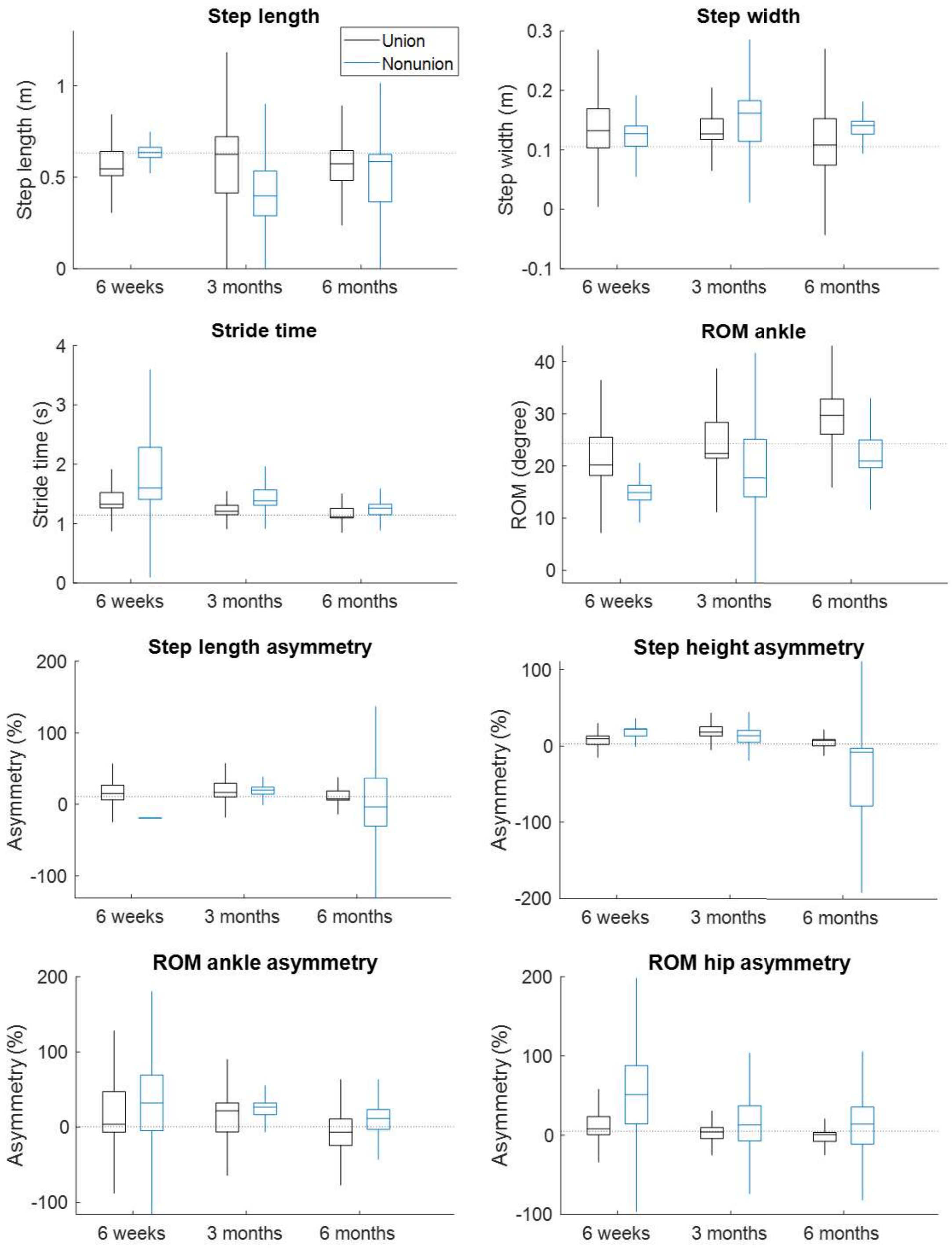

Supplementary figure 2. Boxplots of a subset of the gait parameters of the tibial shaft fracture union (black) and nonunion (blue) group at six weeks, three months and six months.
